# Stevens–Johnson syndrome induced by Sintilimab: a case report and literature review

**DOI:** 10.3389/fonc.2025.1568316

**Published:** 2025-05-22

**Authors:** Huan Kuang, DongBei Huang, ChuXiang Hu, LiPing Gong, ZiYu Yu, XunJin Zhu, HongRong Lan, Gang Huang

**Affiliations:** ^1^ Department of Postgraduate, Jiangxi University of Chinese Medicine, Nanchang, Jiangxi, China; ^2^ Dermatology Department, Affiliated Hospital of Jiangxi University of Chinese Medicine, Nanchang, Jiangxi, China

**Keywords:** Stevens-Johnson syndrome, PD-1 inhibitor, Sintilimab, case report, literature review

## Abstract

**Background:**

Skin diseases induced by Sintilimab, a programmed cell death protein-1 (PD-1) inhibitor, are rare, with only 28 cases reported. We provide a literature review on skin diseases associated with Sintilimab and summarize the patient’s primary disease, duration of Sintilimab use, treatment, and disease progression. This study aims to improve understanding of Stevens–Johnson syndrome (SJS) induced by this monoclonal antibody and its treatment strategies.

**Case description:**

We report a case of SJS induced by Sintilimab in a patient treated at our hospital. The patient exhibited widespread erythema, papules, and vesicles, accompanied by mucosal erosion and exudation in the oral cavity, eyes, urethral orifice, and perianal region. The patient was immediately treated with intravenous methylprednisolone sodium succinate (40 mg/day), antihistamines, and supportive care, including fluid replacement and wound care. His symptoms gradually improved, and he was discharged after 20 days. At the 6-month follow-up, he remained stable, with no recurrence of symptoms.

**Conclusions:**

Although severe drug rash, including SJS, caused by PD-1 inhibitors is relatively uncommon, its underlying molecular pathogenesis remains unclear. Physicians should remain vigilant regarding potential adverse reactions when prescribing Sintilimab. If severe reactions occur, discontinuation of chemotherapy and immediate administration of adequate corticosteroids with symptomatic support can help reduce morbidity and mortality.

## Introduction

PD-1 inhibitors are a class of immune checkpoint inhibitors (ICIs) widely used in the treatment of various malignancies. These inhibitors disrupt the interaction between PD-1 and PD-L1, thereby blocking the binding of immune cells to tumor cells and activating the anti-tumor activity of lymphocytes (1). Currently, these drugs are widely used in the treatment of melanoma (2), non-small cell lung cancer (NSCLC), hepatocellular carcinoma, and other malignancies. However, their immune-modulating effects can lead to excessive immune activation, resulting in multi-system adverse reactions, particularly cutaneous toxicities (3). Skin-related adverse effects occur in approximately 40% of cases and include pruritus, vitiligo, psoriasis, capillary hyperplasia (4), erythema multiforme, SJS, and TEN. The latter two types, although rare, are severe and potentially fatal. This report presents a case of severe SJS induced by the use of a PD-1 inhibitor (Sintilimab) and provides a literature review of similar cases.

## Case description

**Figure 1:**
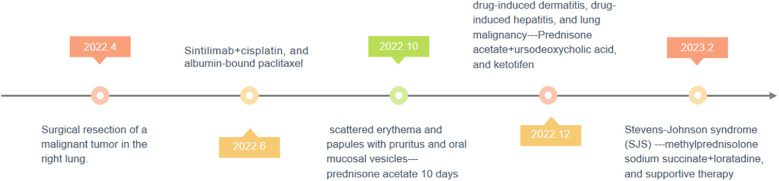
Timeline of clinical course and treatment in a patient receiving Sintilimab.

**Figure 2:**
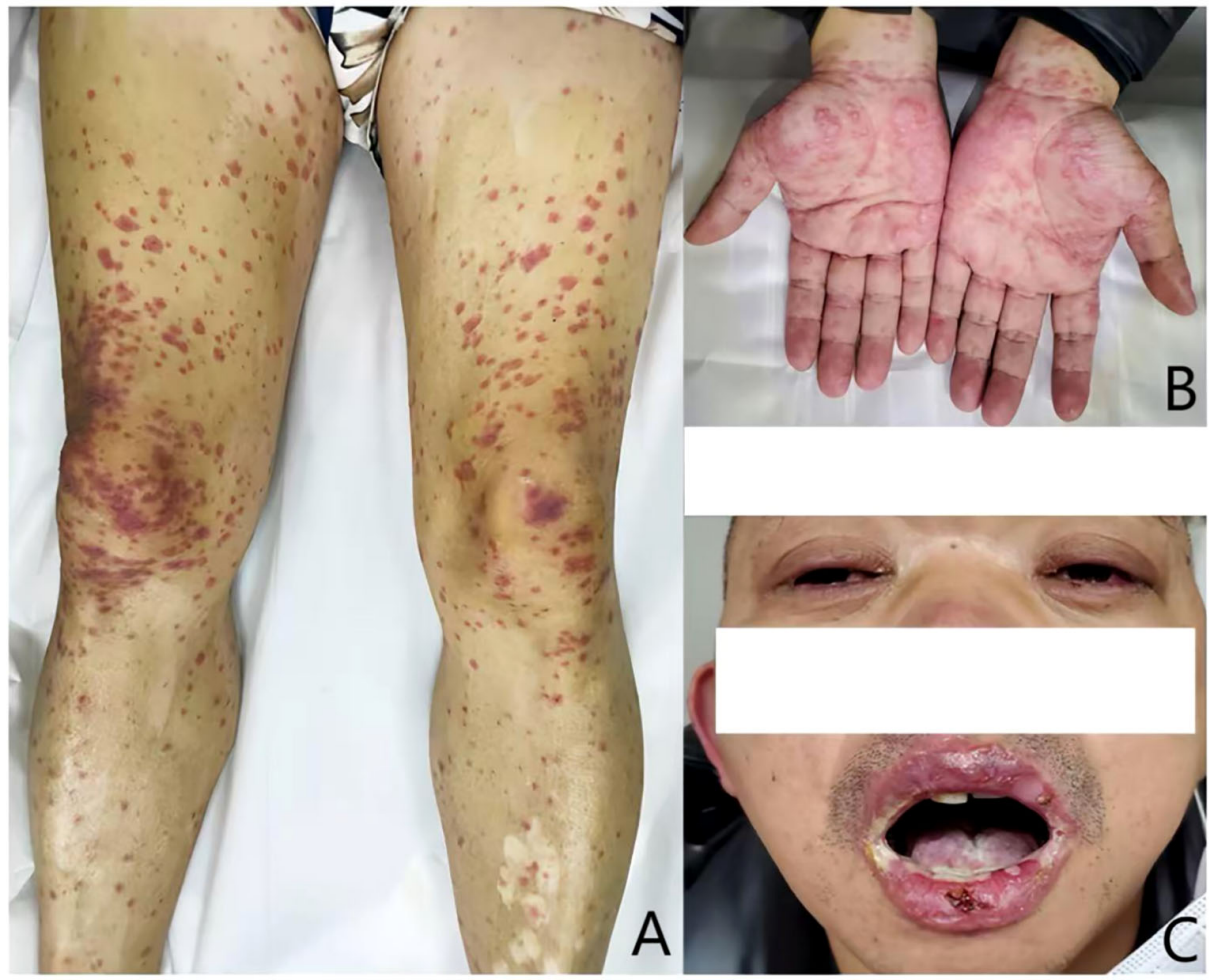
Skin and mucosal damage before antiallergic therapy: **(A)** generalized edematous erythema on limbs; **(B)** the skin lesions exhibited a target-like appearance; and **(C)** erosion of the oral mucosa and blephar.

On 2 February 2023, a 49-year-old male presented with widespread erythema, blisters, itching, and pain that had persisted for 3 months and worsened over the past 3 days. In April 2022, he underwent surgery for a malignant tumor of the right lung; pathological examination confirmed adenosquamous carcinoma of the right upper lung, predominantly squamous cell carcinoma with 10% adenocarcinoma. On 8 June, 2022, he commenced chemotherapy with Sintilimab (200 mg), cisplatin (90 mg), and albumin-bound paclitaxel (300 mg). After the fourth cycle of chemotherapy in October 2022, he developed scattered erythema and papules with pruritus and oral mucosal vesicles. His oncologist recommended discontinuation of chemotherapy and initiated oral prednisone acetate (30 mg/day). Ten days later, as the presentation improved, the patient stopped the drug of prednisone, and erythema and vesicles gradually developed all over the body. In December 2022, he was hospitalized again and diagnosed with drug-induced dermatitis, drug-induced hepatitis, and lung malignancy. Prednisone acetate, ursodeoxycholic acid, and ketotifen were prescribed, yet the specific drug dose was unknown. Sintilimab was discontinued, and the patient was discharged upon symptom resolution. Three days ago, on 2 February 2023 (**Figure 1**), he experienced a sudden exacerbation of erythema, papules, and blisters over his entire body. The lesions exhibited a target-like appearance, predominantly affecting the limbs. Additionally, the patient presented with erosion of the oral mucosa, urethral orifice, and anal mucosa, and crusting of the lips with yellow discharge. Other symptoms included limitation of mouth opening, pharyngeal pain, dysphagia, redness and swelling of the eyelids, conjunctival congestion, photophobia, tearing, dry mouth, and a bitter taste. There were no signs of fever, joint pain, or chills. The patient had pain during urination, which suggested the involvement of a urinary tract infection. He had a history of diabetes mellitus and lung tumor surgery. Therefore, he was admitted to the Dermatology ward with a diagnosis of erythema multiforme, abnormal liver function, postoperative lung tumor, and type 2 diabetes mellitus (**Figure 2**).

Admission examination showed the following: T, 36.7°C; P, 126 beats/min; R, 20 breaths/min; and BP, 129/90 mmHg. Lung auscultation reveals weakened respiratory sounds. No obvious abnormalities are observed in the heart and abdomen. A 6-cm long surgical scar is visible on the right side of the chest. Neurological examinations are negative. Routine laboratory examinations revealed abnormal levels of neutrophil percentage (81.5%) and lymphocyte percentage (14.1%). Blood chemistry revealed increased gamma-glutamyl transpeptidase (212.4 U/L) and total IgE (113.4I U/mL) levels. Analysis of urine–stool samples showed the presence of ketones (+), sugar (4+), and occult blood (+). Glycated hemoglobin and general bacterial culture showed no significant abnormalities.

**Figure 3:**
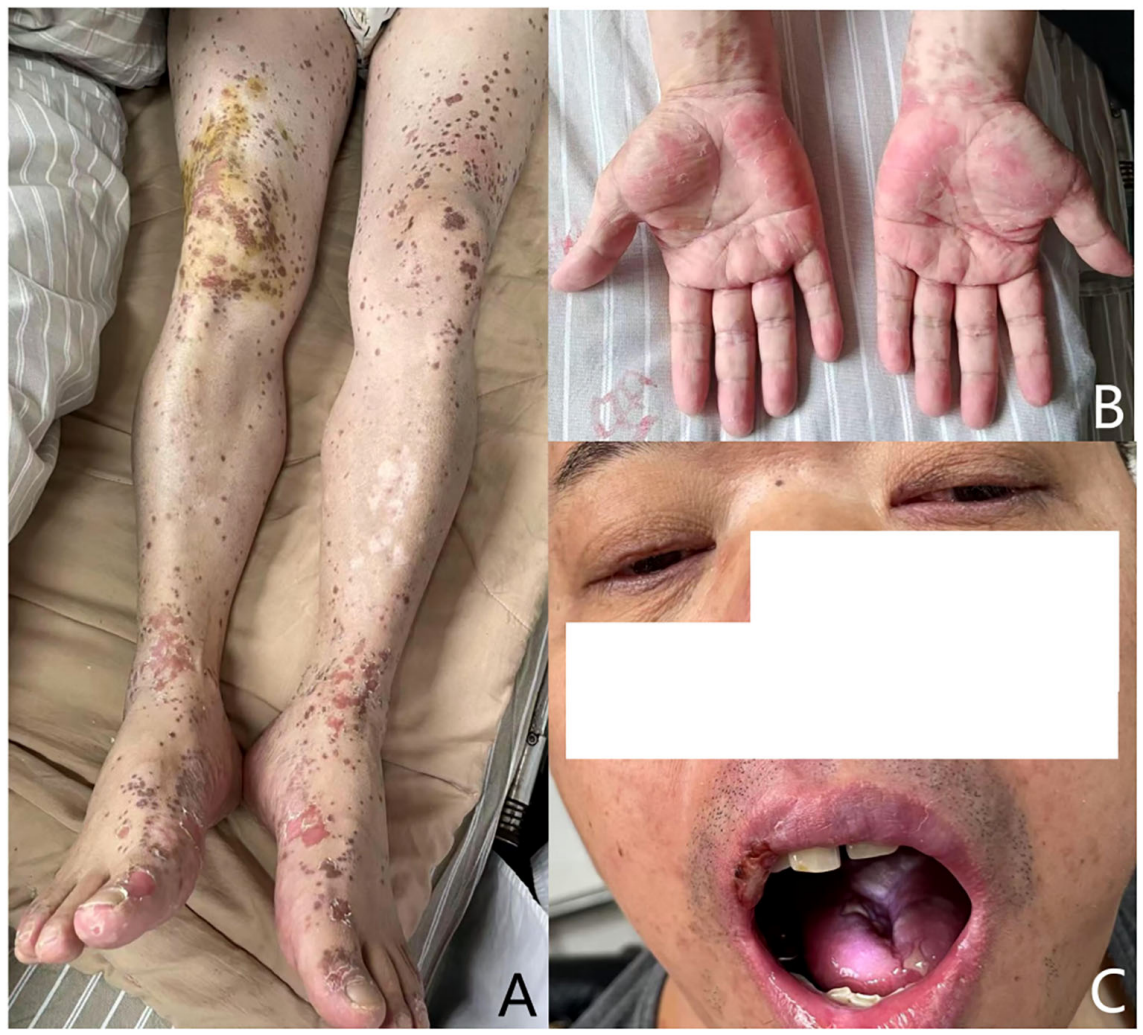
Lesions improved after 14 days’ therapy: **(A)** the vesicular and hemorrhagic lesions on the limbs and trunk had mostly resolved; **(B)** target-like eruption relieved; **(C)** significant improvement in the erosion of the lips and oral mucosa.

Using the Severity-of-Illness Score for Toxic Epidermal Necrolysis (SCORTEN) scoring system (5), the patient’s score of 4 indicated a 62% mortality risk. As this patient was at high risk, routine blood tests, liver and kidney function, blood glucose, electrolytes, and other important indicators were monitored to assess the progression. Sintilimab was suspected as the allergen, and treatment included methylprednisolone sodium succinate (40 mg qd), loratadine (10 mg qd), and supportive therapy (gastric and hepatic protection and electrolyte correction). Skin care treatment was actively applied, including extraction of blisters, mupirocin ointment, and mometasone furoate cream for external use on erythematous spots, 0.9% saline for oral cavity, tobramycin dexamethasone eye drops and erythromycin eye ointment for eye care, avoiding light; within regions of genitalia, perianal area, saline external washing, and external application of human epidermal growth factor gel and mupirocin ointment were used. The patient’s skin erythema subsided, the blisters dried, and crusts were formed, and the erosion in the oral cavity and external genitalia was relieved. As no new lesions developed and the general condition improved, the patient was discharged on 22 February 2023 (**Figure 3**). At the 6-month follow-up, he had not resumed Sintilimab treatment, and no recurrence of symptoms was observed. He also reported that the skin and mucosal symptoms had significantly impacted his daily life. However, after receiving treatment, his condition gradually improved. He expressed satisfaction with the treatment outcome, stating that it exceeded his expectations and helped him regain confidence in his health.

## Literature review

We searched PubMed (https://pubmed.ncbi.nlm.nih.gov/), Embase, Web of Science, Google Scholar (http://scholar.google.com/), WanFang Database, and Chinese National Knowledge Infrastructure (CNKI). Keywords included “sintilimab,” “sintilizumab,” “PD-1 inhibitor,” “tumor,” “tumour,” “skin disease,” “Dermatological disease,” and “Stevens–Johnson syndrome” published in English or Chinese language. Inclusion criteria included the following: (1) study types include case reports, case series, retrospective studies, prospective studies, systematic reviews, and meta-analyses; (2) literature published in English and Chinese to ensure coverage of relevant studies from major medical databases; and (3) studies focusing on cutaneous adverse reactions induced by Sintilimab. Exclusion criteria included the following: (1) conference abstracts or discussions; (2) literature for which full text is unavailable; (3) case reports or studies unrelated to Sintilimab; and (4) cases without a confirmed diagnosis of cutaneous adverse reactions.

A total of 28 cases of Sintilimab-induced cutaneous adverse reactions were documented (**Table 1**), including 14 cases of TEN and severe erythematous drug eruptions, three herpetic pemphigoid, one herpetic dermatitis, four vitiligo, one purpura-like vasculitis, one eczema, one aggravated psoriasis, one lichen-like dermatitis, one pruritus, and one unexplained skin immune-related adverse reactions. There were 20 male patients (71.4%) with the age of 24–82 (64.7 ± 12.2) years, and eight women (28.6%) with 27–72 (53 ± 15.8) years. The most cases with primary disease were lung cancer (10 cases), followed by esophageal cancer (three cases), intestinal cancer (three cases), hepatocellular carcinoma (two cases), nasopharyngeal carcinoma (two cases), and one case of gastric cancer, lymphoma, gallbladder cancer, cervical cancer, malignant melanoma, renal carcinoma, thymus carcinoma, and gingival carcinoma, respectively. Most patients (19/28, 67.86%) had adverse reactions within 50 days after the first administration, and the longest incubation period is 2 years. More than half of the patients had erythema and blisters as skin lesions (64.3%). As treatment, glucocorticoids were routinely used, besides immunoglobulins (37.5%), antibiotics (41.7%), and antihistamines (33.3%) were commonly used (**Table 1**). The administration of the antitumor regimen was ceased in a total of 18 patients, continued in two cases, and information unknown in eight cases. After treatment, 22 patients’ skin damage improved or cured, five patients died, and one patient was lost. Of the five died cases, three died from SJS and toxic epidermal necrolysis.

**Table 1:** Clinical details of the 28 cases with adverse skin reactions due to Sintilimab.

Case no.	Gender	Age (year)	Primary disease	Immunotherapy program	Time of onset	Cutaneous adverse events	Treatment	Discontinuation/initiation	Outcome
1 (19)	M	58	Esophageal cancer	Sintilimab	Cycle 3	SJS	Methylprednisolone, immunoglobulin, hemoperfusion	Unspecified	Improved
2 (20)	M	66	Sigmoid colon cancer	Sintilimab	cycle 1 day 2	SJS	methylprednisolone, loratadine	Ceased	Improved
3 (21)	M	59	Non-small cell lung cancer	Sintilimab	after cycle 3	TEN	Methylprednisolone, Levofloxacin	Ceased	Improved
4 (22)	F	70	Gastric adenocarcinoma	Sintilimab, oxaliplatin	Cycle 1 day 10	TEN	Methylprednisolone, immunoglobulin, adalimumab	Unspecified	Improved
5 (23)	M	65	NK/T-cell lymphoma	Sintilimab, gemcitabine, oxaliplatin	After cycle 4	TEN	Methylprednisolone, cetirizine, immunoglobulin, piperacillin tazobactamna	Ceased	Improved
6 (24)	F	72	Gallbladder cancer	Sintilimab, anilotinib	Cycle 1 day 14	TEN	Methylprednisolone, immunoglobulin, albumin	Ceased	Improved
7 (25)	M	53	Primary rectal cancer	Sintilimab, bevacizumab	After Cycle 11	TEN	Prednisone tablets, methylprednisolone, cetirizine	Ceased	Improved
8 (11)	M	76	Lung adenocarcinoma	Sintilimab, paclitaxel liposomes, carboplatin	Cycle 1 day 24	TEN	Methylprednisolone, prednisone tablets, immunoglobulin	Ceased	Improved
9 (26)	F	27	Nasopharyngeal carcinoma	Sintilimab, albumin paclitaxel, cisplatin	14 days after cycle 4	SJS/TEN	Methylprednisolone, imipenem, immunoglobulin	Unspecified	Unknown
10 (27)	M	66	Lung adenocarcinoma	Sintilimab, pemetrexed, cisplatin	7 days after cycle 1	SJS/TEN	Methylprednisolone, loratadine, dexamethasone, cefoperazone sulbactam sodium, vancomycin, immunoglobulin	Ceased	Improved
11 (28)	M	78	Intrahepatic cholangiocarcinoma	Sintilimab, lenvatinib	18 days after cycle 1	TEN	Gammaglobulin, methylprednisolone	Ceased	Died
12 (29)	F	38	Squamous cell carcinoma of the cervix	Sintilimab, albumin paclitaxel, cisplatin	Cycle 3	TEN	Diphenhydramine, methylprednisolone, immunoglobulin	Ceased	Died
13 (30)	M	69	Lung squamous carcinoma	Sintilimab, paclitaxel, carboplatin	Cycle 1 day 2	TEN	Methylprednisolone, gammaglobulin, itraconazole	Unspecified	Died
14 (31)	M	82	Squamous cell carcinoma of thymus	Sintilimab	cycle 1 day 7	TEN	Methylprednisolone, immunoglobulin, prednisone tablets, broad-spectrum antibiotics	Ceased	Died
15 (32)	M	70	Adenocarcinoma of the bowel	Sintilimab, furaquitinib	Cycle 1–5 months	Herpetic pemphigoid	Methylprednisolone	Continued	Improved
16 (33)	M	73	Malignant melanoma of the heel	Sintilimab	Cycle 7	Herpetic pemphigoid	Minocycline, nicotinamide	Ceased	Improved
17 (34)	M	55	Lung adenocarcinoma	Sintilimab, pemetrexed, carboplatin	Cycle 3, 8 days	Herpetic pemphigoid	Piperacillin tazobactam, fusidic acid cream, cetirizine, granulocyte colony-stimulating factor	Ceased	Improved
18 (35)	M	24	Renal cell carcinoma	Sintilimab, axitinib	Approx. 2 years after cycle 1	Dermatitis herpetiformis	Methylprednisolone, dimethylaminotetracycline, nicotinamide	Ceased	Improved
19 (36)	F	51	Nasopharyngeal cancer	Paclitaxel, cisplatin, Sintilimab	26 days after cycle 3	Vitiligo	Compound glycyrrhizin, Paeonia lactiflora, halomethasone, tacrolimus	Unspecified	Improved
20 (37)	M	73	Lung adenocarcinoma	Sintilimab, bevacizumab	cycle 8	Vitiligo	No relevant treatment seen	Unspecified	Improved
21 (38)	M	Elderly	Gingival squamous cell carcinoma	Sintilimab, albumin paclitaxel, tiglio	After cycle 2	Vitiligo	No relevant treatment seen	Unspecified	Improved
22 (39)	F	46	Lung adenocarcinoma	Pemetrexed, Sintilimab	14 days into cycle 1	Vitiligo exacerbated	No relevant treatment seen	Continued	Improved
23 (40)	M	64	Esophagogastric cancer	Sintilimab	Cycle 1 day 5	Purpura angiitis	Diphenhydramine, methylprednisolone	Ceased	Died
24 (41)	F	65	Squamous cell lung cancer	Sintilimab, albumin paclitaxel, cisplatin	Cycle 3	Psoriasis recurrence	Pavoline, anti-silver granules and narrow-spectrum ultraviolet radiation	Ceased	Improved
25 (42)	M	67	Squamous lung cancer	Sintilimab, cisplatin, albumin paclitaxel	Cycle 2, day 13	Eczema	Thalidomide, avastin, methylprednisolone, piperacillin tazobactamna combined with levofloxacin	Ceased	Improved
26 (43)	M	71	Squamous non-small cell lung cancer	Sintilimab	Cycle 5, day 7	Tinea versicolor	Dermatitis Modified Wilson’s broth	Ceased	Improved
27 (44)	M	65	Hepatocellular carcinoma	Sintilimab	cycle 2 day 14	Alopecia Universalis	No relevant treatment seen	Unspecified	Improved
28 (45)	F	55	Esophageal squamous cell carcinoma	Sintilimab	Cycle 1–14 days	Skin immune-related adverse reactions	Methylprednisolone, levocetirizine, chlorpheniramine maleate tablets, prednisone tablets	Ceased	Improved

M, male; F, female; SJS, Stevens–Johnson syndrome; TEN, toxic epidermal necrolysis.

## Discussion

SJS is a rare severe drug-induced hypersensitivity reaction affecting the skin and mucous membranes (6). The disease usually starts with flu-like symptoms, i.e., fever, cough, epipephysitis, rhinitis, oral ulcers, and arthralgia (7), followed by a painful rash of erythema, papules, urticaria, and purpura. Skin lesions can occur on any part of the body and are often associated with mucosal lesions, including mouth, lips, eyes, respiratory system, digestive system, and urogenital tracts (8). Diffuse purplish-red or dark-red plaques, and the erythema gradually merge into a patch, further forming loose blisters and massive epidermal exfoliation. Severe infection, i.e., septicemia is the most cause of death (9). The SCORTEN scoring is used to predict the risk of mortality, and this patient got a score of 4 and had a high death risk (62%) (10). Luckily, with active treatment of systemic glucocorticoids and adjuvant therapy, he was discharged from the hospital with a full recovery. In comparison to Li et al. (11), which reported a 76-year-old lung adenocarcinoma patient with SJS following Sintilimab treatment, our case involves a younger (49-year-old) patient with adenosquamous carcinoma. Notably, they identified PD-L1 expression in the glands and basal layer of the skin, which may have contributed to localized immune activation leading to SJS. Our case, although lacking immunohistochemical analysis, suggests that tumor histology and patient-specific factors might influence the clinical presentation and severity of SJS induced by PD-1 inhibitors. This highlights the need for further studies examining the immunopathological mechanisms underlying SJS across different tumor types.

The pathogenesis of severe SJS is not yet fully understood and T-lymphocyte-mediated immune reaction causing destruction of the keratinocytes-expressing foreign antigens is regarded as the primary cause of blistering and edematous erythema (12). Drugs are the most common causes, with penicillins, sulfonamides, non-steroidal anti-inflammatory drugs (NSAIDs), carbamazepine, allopurinol, and immunosuppressants as the commonest drugs for SJS (13). Genetic studies have demonstrated significant associations between specific human leukocyte antigen (HLA) alleles and an increased risk of severe cutaneous adverse reactions. For example, HLA-B15:02 is linked to carbamazepine-induced SJS/TEN in Han Chinese populations, while HLA-B58:01 is associated with allopurinol-induced severe cutaneous reactions. Although genetic testing was not performed in our case, the potential role of HLA polymorphisms in PD-1 inhibitor-induced SJS should not be overlooked. Further research into the immunogenetic mechanisms underlying SJS/TEN could pave the way for predictive screening strategies to enhance patient safety (14). Anticancer drugs like immune checkpoint inhibitors are also reported to cause SJS/TEN. As PD-1/PD-L1 inhibitors are increasingly used in clinical, and their potential side effects should be of concern. Cancer patients might be at a higher risk of developing severe blistering and toxic reactions, due to the nature of neoplastic diseases more exposure to a line of anticancer drugs, and disruption of the immune system (15). These anticancer drugs may trigger an abnormal cytotoxic T-lymphocyte response, predisposing them to SJS/TEN.

While our study focuses on the acute immune-mediated pathogenesis of SJS, it is important to differentiate this from chronic epigenetic-driven skin stress responses, such as ultraviolet (UV)-induced photoaging. Song et al. (16) demonstrated that carnitine acetyltransferase (CRAT) downregulation via promoter hypermethylation leads to increased matrix metalloproteinase-1 (MMP-1) expression, which contributes to collagen degradation and chronic skin damage. In contrast, SJS is characterized by rapid, immune-mediated keratinocyte apoptosis rather than gradual fibroblast dysfunction. This distinction underscores the diverse molecular mechanisms governing skin injury—one being a slow, epigenetically driven process (photoaging), and the other a swift, cytotoxic event mediated by immune activation (SJS).

Based on our analysis, when patients receiving anticancer therapy with Sintilimab develop erythema, and maculopapular rashes, particularly vesicular and mucosal lesions, clinicians should strongly suspect SJS and immediately discontinue the medication. Extensive epidermolysis can lead to multi-organ damage, including fluid and electrolyte imbalance, thermoregulatory dysfunction, hepatic and renal impairment, and secondary infections. In addition to anti-allergy medications, comprehensive and supportive treatment is essential, including patient isolation, continuous monitoring of vital signs, meticulous wound care (cleaning necrotic vesicles and scabs), and appropriate anti-infective therapy. Furthermore, rehydration and nutritional support are crucial for recovery. Among the systemic treatment options are intravenous immunoglobulins, glucocorticoids, and cyclosporine; however, glucocorticoids remain the first-line treatment (17). Studies have demonstrated that early and adequate systemic glucocorticoid administration effectively controls disease progression and significantly reduces mortality compared to non-users (18). In this case, the patient was treated with methylprednisolone sodium succinate symptomatically after being admitted to the hospital, and the clinical outcome was remarkable. Moreover, in our review analysis, all the patients received glucocorticoid therapy.

## Conclusion

Sintilimab, a PD-1 inhibitor, was launched in China in December 2018 with a relatively short period of use. There are few reports of its adverse effects up to now, and therefore, more caution should be exercised with these new drugs. In this review, we analyze the significant association between “skin eruption” and “Sintilimab” and find that more than half of the cases presented as SJS/TEN. Therefore, clinicians should be aware of the potential for severe immune-related dermatological toxicity when administering PD-1 inhibitor reactions. If skin erythema, papules, or vesicles occur during anticancer therapy with Sintilimab, the chemotherapy regimen should be immediately discontinued, and an alternative treatment strategy should be promptly considered. Adequate systemic glucocorticosteroid therapy should be initiated to mitigate inflammatory and toxic reactions, which is essential for infection prevention and disease control. Supportive care also plays a crucial role in reducing morbidity and mortality associated with severe adverse reactions.

## Data Availability

The original contributions presented in the study are included in the article/**Supplementary Material**. Further inquiries can be directed to the corresponding author.
